# The Regulatory Role of m^6^A Modification in the Function and Signaling Pathways of Animal Stem Cells

**DOI:** 10.3390/cells15020181

**Published:** 2026-01-19

**Authors:** Xiaoguang Yang, Yongjie Xu, Suaipeng Zhu, Mengru Wang, Hongguo Cao, Lizhi Lu

**Affiliations:** 1 College of Animal Science and Technology, Anhui Agricultural University, Hefei 230036, China; 2 Institute of Animal Husbandry and Veterinary Science, Zhejiang Academy of Agricultural Sciences, Hangzhou 310021, China; 3 Anhui Province Key Laboratory of Local Livestock and Poultry Genetic Resource Conservation and Bio-Breeding, Anhui Agricultural University, Hefei 230036, China

**Keywords:** m^6^A, stem cells, transcription factors, reprogramming, signal pathway

## Abstract

As a type of cell with self-renewal ability and multi-directional differentiation potential, stem cells are closely related to their functions, such as reprogramming transcription factors, histone modifications, and energy metabolism. m^6^A (N^6^-methyladenosine modification) is one of the most abundant modifications in RNA, and dynamic reversible m^6^A modification plays an important role in regulating stem cell function. This review moves beyond listing isolated functions and instead adopts an integrated perspective, viewing m^6^A as a temporal regulator of cellular state transitions. We discuss how m^6^A dynamically regulates stem cell pluripotency, coordinates epigenetic and metabolic reprogramming, and serves as a central hub integrating key signaling pathways (Wnt, PI3K-AKT, JAK-STAT, and Hippo). Finally, using somatic reprogramming as an example, we elucidate the stage-specific role of m^6^A in complex fate transitions. This comprehensive exposition not only clarifies the context-dependent logic of m^6^A regulation but also provides a precise framework for targeting the m^6^A axis in regenerative medicine and cancer therapy.

## 1. Introduction

Stem cells, as a type of cell with multi-directional differentiation potential and self-renewal ability, can differentiate into cells of a specific tissue type [1]. Stem cell function is closely related to reprogramming transcription factors [2], histone modifications [3], and energy metabolism [4]. With the continuous development of life science research, human understanding of stem cells is gradually deepening, and research achievements related to stem cells are playing an increasingly important role in disease treatment, regenerative medicine, and other fields [5].

In recent years, post-transcriptional RNA modifications have emerged as a form of epigenetic regulation extensively involved in gene expression control. Nucleotide modifications are present in various RNA transcripts, both coding and non-coding [6]. m^6^A modification, as an important RNA modification, participates in regulating mRNA processing and metabolism in various biological processes [7].

In 1974, m^6^A was first discovered to be the main form of mRNA methylation in mammals. As the most common and abundant post-transcriptional RNA modification in eukaryotic cells, it accounts for over 50% of all methylated ribonucleotides in total RNA in cells, with an average of 3–5 m^6^A sites per mRNA [8,9]. The m^6^A modification is distributed in the RRACH consensus sequence (where R = A/G and H = A/C/U), mainly enriched in the termination codon and 3′ untranslated regions (3′ UTRs) [10]. m^6^A modification is dynamically regulated by the interactions between various m^6^A-specific protein families, known as methyltransferases (writers), demethylases (erasers), and readers [11]. m^6^A modification affects almost every step of RNA metabolism, including processes such as alternative splicing (AS), stability regulation, degradation, translation, and nuclear export [12]. This in turn drives many biological processes, including circadian rhythms, T cell differentiation, stem cell renewal and differentiation, the epithelial–mesenchymal transition, etc. [13,14]. In stem cells, m^6^A controls the functional changes in stem cells by regulating processes such as RNA splicing, maturation, stability, translation, and localization [15]. Research has found that m^6^A plays an important role in regulating stem cell function, such as regulating reprogramming transcription factors [16], histone modifications [17], energy metabolism [18], and reprogramming efficiency of induced iPSCs (pluripotent stem cells) [19].

m^6^A modification plays a central role in the development of individual organisms on a cellular basis. This review introduces m^6^A modification and focuses on the regulatory role of m^6^A in stem cell pluripotency maintenance, histone modification, energy metabolism, and iPS cell induction. At the same time, the regulatory role of m^6^A in key signaling pathways of stem cells was elucidated. This review helps to better understand RNA modification and its mechanisms in the field of life sciences and provides prospects for future research on cell proliferation and human regenerative medicine.

## 2. m^6^A Modification

m^6^A modification, as a highly reversible process, regulates nearly every step of RNA metabolism [12]. It is dynamically installed, removed, and interpreted by writers, erasers, and readers, respectively. Extensive biochemical and structural studies have established that the METTL3-METTL14 heterodimer constitutes the catalytic core of the m^6^A methyltransferase complex (MTC), with *METTL3* providing the catalytic site and *METTL14* stabilizing RNA substrate binding [20,21]. This core complex invariably associates with adaptor proteins such as *WTAP*, which is essential for directing m^6^A deposition to proper genomic loci. The MTC modulates cellular functions in HeLa cells, leukemia cells, pancreatic cancer cells, and others, playing vital roles in related disease pathogenesis and treatment [22,23]. Demethylases, including *ALKBH5* and *FTO*, remove m6A marks [24]. Readers, primarily the YTH domain family proteins (*YTHDF* and *YTHDC* classes), along with *HNRNP* and *IGF2BP* proteins, recognize and execute the functional outcomes of m6A [11,25].

**Figure 1:**
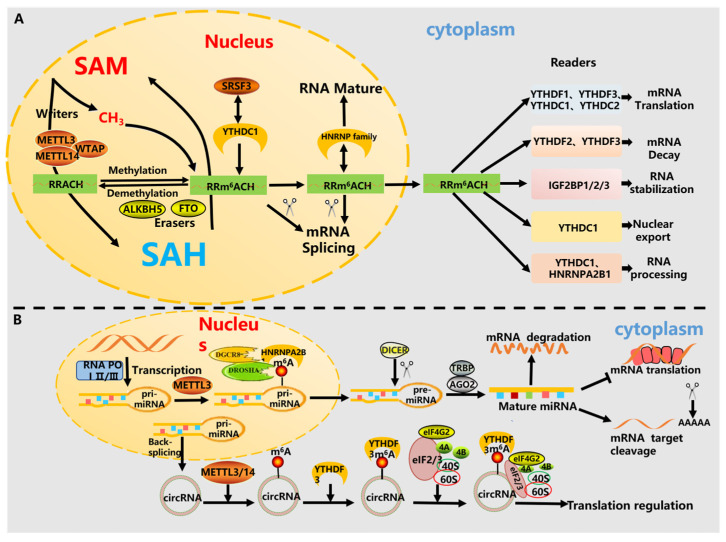
(**A**) Dynamic m^6^A modification of mRNA. The schematic depicts the reversible m^6^A modification cycle. Writers (e.g., the *METTL3/14* complex) deposit m^6^A marks using S-adenosylmethionine (SAM) as the methyl donor [21]. Erasers (*FTO* and *ALKBH5*) remove these marks. Reader proteins (e.g., *YTHDF* family, *YTHDC* family, *IGF2BPs*, and *HNRNPs*) recognize m^6^A and determine the functional outcome for the modified mRNA, influencing its splicing, export, stability, or translation [11,24,25]. (**B**) m^6^A modification of non-coding RNAs. Beyond mRNA, m^6^A also regulates the biogenesis and function of non-coding RNAs. *METTL3* facilitates the processing of primary microRNAs (pri-miRNAs) into mature miRNAs. Additionally, m^6^A modification on circular RNAs (circRNAs) can promote their cap-independent translation through reader protein (e.g., *YTHDF3*)-mediated recruitment of translation initiation factors [37,38].

m6A regulates mRNA alternative splicing (AS), stability, degradation, translation, and nuclear export [26] (Figure 1A). The methyltransferase localizes to nuclear speckles [27]. *WTAP* promotes METTL3/14 accumulation in speckles to influence AS, while *FTO* regulates AS by preventing *SRSF2* recruitment [28,29]. *YTHDC1* modulates AS by binding splicing factors SRSF3/10 within nuclear speckles. Upon export to the cytoplasm, m6A-modified transcripts are recognized by cytoplasmic readers, enhancing RNA export efficiency—a process relevant to stem cell disease therapeutics [30,31]. *IGF2BPs* stabilize mRNA by recruiting *HuR, MATR3*, and *PABPC* [32]. *YTHDF2* recruits the *CCR4-NOT* deadenylase complex to promote mRNA decay, whereas *YTHDF1* facilitates translation by recruiting eIF3 [33,34].

m6A also modulates non-coding RNAs. *METTL3* and *HNRNPA2B1* interact with *DGCR8* and *DROSHA* to regulate pri-miRNA processing into mature miRNA [35,36]. circRNA, generated through backsplicing of pre-mRNA, lacks a 5′ cap and often relies on m6A modification within its 5′UTR for cap-independent translation, a process regulated by *YTHDF3* [37,38] (Figure 1B). Furthermore, YTHDF proteins influence embryonic development, stem cell fate, adipogenesis, and tumorigenesis, highlighting their potential as predictive biomarkers and therapeutic targets [39].

The functions of m^6^A “read–write–erase” proteins may extend beyond their classical roles. A key concept is distinguishing between actions dependent on their enzymatic or binding activities versus those dependent on their scaffold/structural protein functions.

m^6^A-dependent mechanisms: Function directly relies on the protein’s catalytic activity (e.g., *METTL3*’s methyltransferase activity) or binding activity (e.g., *YTHDF2* recognizing m^6^A marks) [40]. Examples include *METTL3*-mediated m^6^A deposition [41] and *YTHDF2*-mediated mRNA degradation following m^6^A recognition [42]. m^6^A-independent mechanisms: Function arises from their capacity to serve as protein interaction platforms or components of chromatin-regulating complexes, independent of enzymatic/binding activity. A classic example is *METTL14*: it regulates H3K27me3 levels by interacting with the PRC2 complex and recruiting *KDM5B*, a function independent of its methyltransferase activity [43,44]. This discovery critically expands the functional paradigm of the core “writer” proteins, revealing that they can serve as scaffold molecules to directly bridge RNA methylation with chromatin remodeling. It underscores a key caution in the field: the phenotypic consequences of depleting an m^6^A factor may not always be attributable to the loss of m^6^A marks per se but could arise from disrupting its non-catalytic, structural roles. Similarly, *YTHDC2* recruits the histone methyltransferase *MLL1* in cancer stem cells [45], a role that may also be partially independent of its reading function.

## 3. m^6^A Modification and Stem Cell Pluripotency

The maintenance and exit from pluripotency are fundamental to stem cell biology. This section addresses a pivotal question: How does m^6^A, often perceived as a stabilizer of pluripotency, also facilitate the timely transition to differentiation? We will first delineate the canonical paradigm where m^6^A safeguards pluripotency by stabilizing transcripts of core factors like *NANOG*. We will then contrast this with emerging evidence that m^6^A-mediated decay of a subset of pluripotency transcripts is essential for differentiation priming. This duality highlights m^6^A’s role as a dynamic modulator rather than a static lock, with non-coding RNAs (e.g., *linc1281*) serving as key intermediaries in this process.

The maintenance of pluripotency is crucial for stem cell function, governing their proliferation and differentiation [46]. This state requires high levels of transcriptional and ribosomal activity [47], processes that are centrally regulated by m^6^A RNA modification [15]. m^6^A safeguards pluripotency primarily by stabilizing the mRNAs of key pluripotent factors [48,49]. It should be noted that the role of m^6^A in pluripotency is context-dependent and not a single-mode phenomenon. Contrary to the “stability model,” Geula et al. found that m^6^A methylation is essential for the timely release of initial pluripotency and promotes differentiation by facilitating the turnover of certain initial pluripotency transcripts [49]. This apparent paradox suggests that m^6^A does not simply “lock in” a pluripotent state but rather functions as a dynamic modulator of RNA fate, where its outcome (stabilization vs. decay) is determined by specific cellular contexts, transcript identities, and the complement of bound reader proteins. *ERK* phosphorylates *METTL3* at the S43/S50/S525 site, phosphorylates *WTAP* at the S306/S341 site, deubiquitinates through *USP5*, and finally produces stable m^6^A methyltransferase complexes. The lack of *METTL3/WTAP* phosphorylation reduces the decay of m^6^A-labeled pluripotent factor transcripts and maintains the pluripotency of mESC (mouse embryonic stem cells) [50]. This regulatory axis of *METTL3* phosphorylation is also functional in other stem cell contexts, such as dental pulp and pancreatic cancer stem cells [51].

*METTL3* is a key protein regulating stem cell pluripotency [52]. A pivotal yet context-sensitive finding is that depletion of *METTL3* in mouse embryonic stem cells (mESCs) can, counterintuitively, lead to increased *Nanog* expression and impede differentiation [53]. This contrasts with its general role in stabilizing pluripotency transcripts, underscoring that *METTL3*’s function is not monolithic. It suggests that in naive pluripotency, *METTL3* may preferentially target a subset of transcripts for degradation to prime cells for differentiation, a function that appears to be highly dependent on the specific cellular state. The m^6^A machinery intricately controls core pluripotency factors: the methyltransferase complex (MTC) interacts with *SMAD2/3* [54], while the demethylase *ALKBH5* partners with *ZFP217* to fine-tune *Nanog* expression [55]. Furthermore, readers like *IGF2BPs* stabilize the transcripts of *OCT4* and *SOX2* by binding to their CDS and 3’UTRs, directly reinforcing the pluripotent state [32,56].

Beyond canonical mRNA targets, m^6^A modification regulates stem cell pluripotency through select long non-coding RNAs (lncRNAs) that function primarily in the cytoplasmic regulatory layer [57]. A key example is *linc1281*, whose m^6^A modification enhances its function as a competing endogenous RNA (ceRNA). By sponging pluripotency-inhibitory *let-7* family miRNAs, m^6^A-modified *linc1281* preserves the expression of pluripotency factors, thereby maintaining mESC identity [58]. Other lncRNAs like *H19* are also m^6^A targets and may operate in similar ceRNA networks [59]. This regulatory axis intersects with the well-known *let-7/Lin28* negative feedback loop [60], illustrating how m^6^A can modulate existing post-transcriptional circuits to reinforce the pluripotent state. It is important to distinguish this cytoplasmic, miRNA-mediated regulation from the nuclear, chromatin-directed mechanisms of m^6^A discussed in the following section.

Emerging Consensus: A foundational principle is that m^6^A functions as a bidirectional rheostat. It maintains the pluripotent ground state by stabilizing core factor mRNAs (e.g., via *IGF2BPs* [32,56]) but also primes cells for differentiation by promoting the turnover of specific naïve pluripotency transcripts [49]. This duality is essential for state transitions.

Model-Dependent Insights and Gaps: The current mechanistic map is predominantly drawn from mouse embryonic stem cells (mESCs). While the conservation of core writers (*METTL3*) and readers is evident, their precise targets and functional outcomes in human ESCs, adult stem cells, or organoid models remain less defined, representing a critical area for comparative studies.

Key Unresolved Controversy: The field is actively debating the functional logic of cytoplasmic readers, particularly the YTHDF family. Evidence points to both context-specific specialization (e.g., distinct roles for *YTHDF1* and *YTHDF2* in porcine iPSCs [16]) and functional redundancy (e.g., cooperative requirement of *YTHDF2/3* in murine reprogramming [61]). Resolving this “redundancy vs. specificity” paradox is essential for predicting the consequences of m^6^A deposition and for designing targeted interventions.

## 4. m^6^A Modification and Histones

Moving beyond direct mRNA regulation, m^6^A exerts a profound influence on the cellular state by interfacing directly with the epigenome. This section focuses on m^6^A’s role as a critical bridge between RNA metabolism and chromatin dynamics. We will detail how m^6^A modification of chromatin-associated RNAs (carRNAs) and its interaction with histone-modifying complexes (e.g., PRC2 and *SETDB1*) actively sculpt the chromatin landscape. This mechanism is not separate from pluripotency control but is a key effector pathway by which m^6^A stabilizes or alters transcriptional programs, including those governing pluripotency and differentiation.

**Figure 2:**
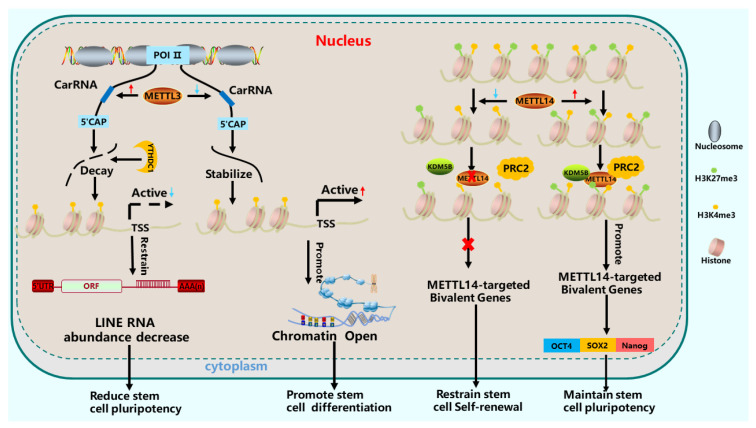
The role of m^6^A modification in histones. *METTL3* controls the stability and expression of carRNA, thereby affecting the activity of chromatin transcription factors [64]. The activity of chromatin transcription factors decreases, and Line RNA degrades, leading to a decrease in stem cell pluripotency. On the contrary, an increase in transcription factor activity enhances chromatin opening and promotes stem cell differentiation [65]. The expression of *METTL14* increases, recruiting PRC2 and *KDM5B7* [43,44]; promoting *METTL14*-targeted bivalent genes; affecting the expression of pluripotency factors such as *OCT4*, *SOX2*, and *Nanog*; and regulating the pluripotency and proliferation of stem cells.

The opening or closing of specific chromatin regions during stem cell proliferation and differentiation is influenced by histone methylation and acetylation [62]. m^6^A modification participates in various histone modifications and regulates chromatin changes [38,63] (Figure 2).

m^6^A modification orchestrates chromatin dynamics in stem cells through multiple, interconnected mechanisms. A primary pathway involves the regulation of chromatin-associated regulatory RNAs (carRNAs). In mESCs, *METTL3*-mediated m^6^A modification of carRNAs (including promoter-/enhancer-associated and repeat RNAs) facilitates an open chromatin state by recruiting chromatin modifiers [64]. Specifically, *METTL3* interacts with *SETDB1* and *TRIM28* to localize to retrotransposons like *IAPs*. This targeting, mediated by the methyltransferase complex (MTC) at *IAP* 5′UTRs, regulates heterochromatin marker deposition and is essential for chromatin integrity and homeostasis [13,66,67].

Beyond carRNAs, m^6^A directly influences histone modification landscapes. *METTL14* interacts with the PRC2 complex and recruits the demethylase *KDM5B* to modulate H3K27me3 levels [17,43,44]. Conversely, the demethylase *FTO* erases m^6^A marks on LINE1 retrotransposon RNA, regulating its abundance and local chromatin condensation [68]. The m^6^A reader *YTHDC1* recognizes m^6^A on *LINE1* and *ERVK* transcripts, recruiting *SETDB1* to enforce transcriptional silencing and maintain mESC homeostasis [69,70,71].

This chromatin-regulatory capacity of m^6^A extends to pathological stem cell contexts. In cancer stem cells, *YTHDC2* recruits the histone methyltransferase *MLL1* to enhance H3K4me3 and oncogene transcription [45]. In acute myeloid leukemia stem cells, *RBFOX2* recognizes m^6^A on carRNAs and recruits *RBM15* to promote repeat RNA methylation, an event that suppresses leukemogenesis and promotes differentiation [72].

The recruitment of m^6^A regulators to specific chromatin loci is not passive but is actively controlled. Extracellular signals can modulate their activity and localization via post-translational modifications. For instance, *ERK*-mediated phosphorylation of *METTL3* and *WTAP*, stabilized by *USP5*-mediated deubiquitination, enhances the integrity and nuclear retention of the methyltransferase complex, potentially directing it to pluripotency gene loci in response to growth factors [50]. Furthermore, sequence-specific transcription factors serve as critical recruiters. The TGF-β pathway effectors *SMAD2/3* physically interact with the MTC, thereby coupling extracellular morphogen signals to m^6^A deposition on key pluripotency transcripts like *Nanog* [54]. Conversely, the transcription factor *ZFP217* can partner with the demethylase *ALKBH5*, forming a complex that is recruited to specific promoters to fine-tune local RNA methylation and histone modification landscapes [55]. These examples illustrate a paradigm where upstream signaling cascades and transcription factors act as the “directors”, guiding the “writers” and “erasers” of m^6^A to precise genomic addresses to execute state-specific epigenetic programs

Core Established Mechanism: It is now well-established that m^6^A on chromatin-associated RNAs (carRNAs) serves as a direct signal for recruiting histone modifiers (e.g., *SETDB1* and PRC2) to specific loci, thereby influencing local chromatin states and transcription [64,66]. This provides a concrete molecular bridge between the epitranscriptome and epigenome.

Contextual Variability and Challenges: While the principle is clear, its functional impact is highly context-dependent. The same reader, *YTHDC1*, can mediate silencing of retrotransposons in mESCs [69] but may have opposite effects in cancer contexts. Furthermore, distinguishing the effects of m^6^A on carRNAs from its concurrent regulation of protein-coding mRNAs at the same locus presents a significant technical and interpretative challenge.

Major Unanswered Question: A central unresolved issue is the hierarchical relationship and potential feedback loops between m^6^A on chromatin and canonical histone marks. Does m^6^A deposition direct histone modification, or do existing chromatin states recruit the m^6^A machinery? Disentangling this causality is crucial for understanding the sequence of events during epigenetic reprogramming.

## 5. m^6^A Modification and Energy Metabolism

Cell fate is closely linked to metabolic status. This section explores how m^6^A, as a core regulator of stem cell metabolism, serves as a critical interface between gene expression and metabolic flux. We will analyze how m^6^A targets key enzymes in glycolysis and oxidative phosphorylation, thereby controlling the metabolic switch between self-renewal and differentiation. Crucially, we highlight that this metabolic regulation is not an isolated function; it directly fuels the epigenetic machinery (e.g., by providing acetyl-CoA for histone acetylation or α-KG for demethylases), thereby creating a cohesive “m^6^A–metabolism–epigenetics” axis that dictates cell fate.

**Figure 3:**
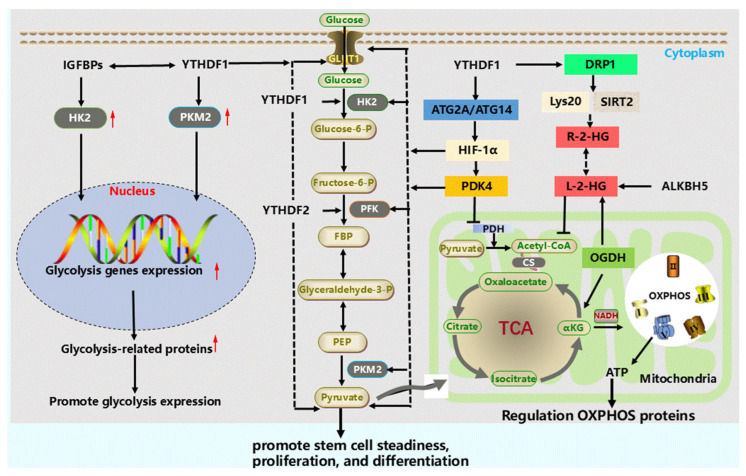
The illustration integrates two interconnected regulatory modules controlled by m^6^A. Left panel, glycolysis promotion: m^6^A reader proteins (e.g., *YTHDF1* and *IGFBPs*) enhance glycolytic flux by upregulating the expression of key glycolytic enzymes (*HK2* and *PKM2*) [18,81] and the transporter *GLUT1*. Right panel, mitochondrial function and OXPHOS modulation: m^6^A also finely tunes mitochondrial function. *YTHDF1* influences mitochondrial dynamics by regulating *HIF-1α* and *DRP1* [80]. The demethylase *ALKBH5* modulates TCA cycle intermediates (e.g., L-2-HG) and acetyl-CoA levels, thereby affecting oxidative phosphorylation (OXPHOS) efficiency and ATP production [86,87]. Critical Integration Point: These two modules are not isolated. Pyruvate from glycolysis feeds into mitochondrial metabolism, while mitochondrial outputs (e.g., acetyl-CoA and α-ketoglutarate) serve as key precursors for epigenetic processes like histone modification. Thus, by coordinating these two metabolic arms, m^6^A shapes the metabolic state that determines stem cell fate, exemplifying the “m^6^A–metabolism–epigenetics” axis.

Energy metabolism directly participates in the maintenance of stem cell pluripotency, stem cell proliferation and differentiation, and affects stem cell function [73,74]. Oxidative phosphorylation and glycolysis are the two main ways for stem cells to obtain ATP [75,76]. m^6^A modification regulates the transcription and translation of key genes involved in oxidative phosphorylation and glycolysis, affecting stem cell energy metabolism [18,77] (Figure 3).

Figure 3 not only illustrates the regulation of metabolic enzymes by m^6^A but also implies its role in reshaping cellular metabolic states to influence stem cell fate. In stem cell biology, the balance between glycolysis and oxidative phosphorylation (OXPHOS) carries distinct functional implications. Active glycolysis (the Warburg effect) typically supports self-renewal and pluripotency maintenance. It not only provides ATP but, more importantly, supplies biosynthetic precursors for cell proliferation. Metabolites such as acetyl-CoA produced from glycolytic flux serve as substrates for histone acetylation, thereby maintaining an open chromatin state and expression of pluripotency genes [78]. Conversely, a shift towards OXPHOS is often associated with differentiation. OXPHOS meets the higher energy demands of differentiated cells more efficiently. Moreover, metabolites from the TCA cycle (e.g., α-ketoglutarate) are essential cofactors for epigenetic modifiers (e.g., TET and KDM families), which activate differentiation-related gene programs [73,79]. Therefore, by precisely regulating the metabolic switches depicted in Figure 3, m^6^A exerts an indirect yet profound influence on stem cell fate decisions.

In the glycolysis of stem cells, the *YTHDF1*/eEF2 complex and *IGFBP3* regulate the 5’UTR of *PDK4* (pyruvate dehydrogenase kinase 4) [80], and *IGFBPs* interact with the 5’/3’UTR of *HK2* (hexokinase 2) and the 3’UTR of *GLUT1* (facilitative glucose transporter), promoting the expression of *HK2* and *GLUT1* [81]. *YTHDF1* regulates the expression of *HK2* and *PKM2* (pyruvate kinase isozyme type M2) [18,82], while R-2-HG upregulates *LDHB* (lactate dehydrogenase B) expression by mediating *FTO* and *YTHDF2*, promoting glycolysis [83].

*METTL3* and *METTL14* jointly regulate *APC* (adenomatous polyposis coli) expression, which increases *c-MYC* (myelocytomatosis viral oncogene homolog) and *PKM2* expression [84]. *METTL3* directly interacts with *ACLY* (ATP citrate lyase) and SLC25*A1* (solute carrier family 25 member 1) [85], affecting glycolysis. Activated glycolysis serves as a switch for controlling histone acetylation, regulating stem cell pluripotency [78]. In mitochondria, *ALKBH5* regulates the TCA (tricarboxylic acid cycle) [86] (Figure 3). *ALKBH5* reduces *DRP1* (Dynamin-related protein 1) methylation levels, inhibits mitochondrial fission, and controls stem cell energy metabolism [87]. In gastric cancer stem cells, *METTL3* modifies the 3’UTR of *NDUFA4* (NADH dehydrogenase 1 alpha subcomplex 4) mRNA, recruits *IGF2BP1* to increase *NDUFA4* mRNA stability, and promotes oxidative metabolism [88]. *NDUFA4* enhances stem cell glycolysis to promote proliferation and differentiation [18]. In iPSCs, OXPHOS and glycolysis are in a balanced state, regulating the proliferation and differentiation of iPSCs through the aminoglycation of *OCT4* and *SOX2*. *YTHDF2* directly regulates *OCT4* and *SOX2* [61,75,89]. The intermediate products produced in OXPHOS and glycolysis are not only important substrates for protein acetylation but also essential for maintaining the chromatin structural characteristics of stem cells [90]. m^6^A regulates energy metabolism in stem cells, which has significant implications and potential for cancer stem cell renewal and differentiation, tumor treatment resistance, tumor metabolism, and tumor therapy [18].

In summary, the profound significance of m^6^A’s regulation of stem cell energy metabolism lies in its direct shaping of the cellular epigenetic landscape. Glycolysis and the TCA cycle serve not only as energy-producing processes but also as supply stations for key metabolic precursors of histone modifications. By regulating the expression of metabolic enzymes, m^6^A precisely controls the flow of these precursors: enhanced glycolysis leads to the accumulation of pyruvate and lactate. Pyruvate can be converted into acetyl-CoA, providing substrates for histone acetylation (e.g., H3K27ac); lactic acid directly drives histone lactylation (e.g., H3K18la). Both modifications correlate positively with chromatin accessibility and pluripotency gene activation [91]. TCA cycle intermediates like α-ketoglutarate (α-KG) serve as essential cofactors for histone demethylases (e.g., KDM) and DNA demethylases *TET*. m^6^A modulates TCA via *ALKBH5* [86] or promotes acetyl-CoA production by stabilizing *PDHA1* mRNA through *YTHDC2* [79], effectively controlling the availability of the “metabolic tool” used to erase repressive epigenetic marks.

Collectively, these findings support a model in which m^6^A modification functions as an upstream regulator within a cohesive “m^6^A–metabolism–epigenetics” axis. By directing metabolic reprogramming and modulating the availability of critical metabolites (such as acetyl-CoA and α-KG), m^6^A installation indirectly governs the dynamics of histone modifications and chromatin states, culminating in fate decisions in stem cells. This axis provides a mechanistic framework for understanding the profound impact of metabolism on cellular reprogramming and pluripotency.

Strong Correlative Evidence: A robust body of data demonstrates that m^6^A regulates key metabolic enzymes and that metabolic shifts (e.g., glycolytic flux) alter the availability of epigenetic cofactors (acetyl-CoA, α-KG) [78,91]. The temporal correlation of these events during fate changes is strongly supportive of an integrated axis.

Causality and Quantitative Gaps: Much of the evidence remains correlative. Precisely how the dynamic, often subtle, changes in m^6^A on individual metabolic enzyme mRNAs quantitatively translate into metabolite pool shifts sufficient to drive global epigenetic changes is not fully mapped. The field lacks dynamic, quantitative flux models that incorporate m^6^A kinetics.

Primary Controversy: The predominant focus has been on how m^6^A-driven metabolism fuels epigenetics. However, the reverse regulation—how epigenetic states or metabolic signals control the m^6^A machinery itself (e.g., via transcription or PTMs of writers/erasers)—is equally important but less explored. The axis is likely bidirectional, and its unidirectional portrayal is an oversimplification.

## 6. m^6^A Modification and Signaling Pathways

Stem cell fate determination represents a coordinated response to intracellular and extracellular signals. Building upon the established role of m^6^A in regulating fundamental cellular states (Section 3, Section 4 and Section 5), this section further explores its higher-order functions as a sophisticated regulator and integrator of key signaling pathways. We will analyze how m^6^A modification of key nodal components (e.g., *APC* in Wnt and *PTEN* in *PI3K-AKT*) dynamically modulates the activity of the Wnt, *PI3K-AKT*, JAK-STAT, and Hippo pathways. A particular focus will be on how m^6^A may orchestrate crosstalk between these pathways by simultaneously targeting shared nodes (e.g., *GSK-3β*), thereby amplifying fate-determining signals and ensuring coordinated cellular responses.

### 6.1. m^6^A Modification and Wnt Signaling Pathway

The Wnt pathway plays a crucial role in stem cell differentiation, proliferation, metabolism, and other processes [92,93]. Key components of this pathway, including *APC*, *GSK-3β*, *AXIN*, and *β-catenin*, are subject to m^6^A modification, which dynamically regulates their expression and function [94].

Studies primarily in colorectal and gastric cancer models have revealed a key oncogenic axis: *METTL3* promotes Wnt/*β-catenin* signaling by recruiting *YTHDF* reader proteins to degrade *APC* mRNA [84]. The consequent accumulation of *β-catenin* upregulates downstream targets like *c-MYC* and *PKM2*, which in these cellular contexts drives a pro-proliferative glycolytic switch. This mechanism provides a clear example of how the m^6^A machinery can be co-opted to fuel pathological stemness, though its activity in normal stem cell homeostasis requires further investigation. Conversely, *METTL3* can also inhibit cell motility by downregulating *c-Met* (cellular mesenchymal–epithelial transition factor), which in turn suppresses the membrane localization of *β-catenin* and its interaction with *E-cadherin*, thereby playing a role in controlling cancer cell proliferation [95].

**Figure 4:**
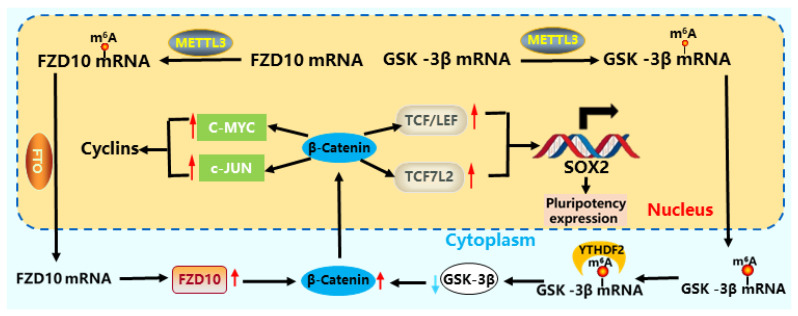
Regulation of m^6^A modification in the Wnt pathway of stem cells. *METTL3*, *FTO*, and *YTHDF2* activate Wnt/*β-catenin* by regulating *FZD10* [96] and *GSK-3β* [101], and the activated Wnt/*β-catenin* translocates into the nucleus. By regulating *C-MYC* and *cJUN*, they affect cyclin, promote the expression of *TCF/LEF* and *TCF7L2*, increase *SOX2* expression, and enhance stem cell pluripotency [100].

The expression of *FTO* and *ALKBH5* is reduced, directly increasing the methylation level of receptor protein *FZD10* mRNA and the expression of *β-catenin*, activating the Wnt pathway, and thereby affecting the gene expression of epithelial ovarian cancer (EOC) [96]. *ALKBH5* binds to *AXIN2* mRNA of the Wnt pathway, causing demethylation [97]. The *FTO* promoter region LEF/TCF binds to *β-catenin*, modifies *MYC*, and maintains stem cell homeostasis [98]. *FTO* serves as a key regulatory factor for Wnt to trigger EMT, altering the 3′ end processing of key mRNA in the Wnt signaling cascade, leading to EMT [99]. *YTHDF1* directly promotes the translation of the Wnt signal transduction effector factor *TCF7L2/TCF4*, enhances the activity of *β-catenin*, and affects the pluripotency of stem cells [100] (Figure 4). *YTHDF2* recognizes and targets m^6^A-modified *GSK-3β* mRNA for degradation, enhances *β-catenin* activity, reduces *YTHDF2* expression, inhibits Wnt/*β-catenin*/Cyclin D1 pathway-related protein expression, leads to G0/G1 phase arrest in cells, and ultimately inhibits CRC cell proliferation [101].

### 6.2. m^6^A Modification and PI3K-AKT Signaling Pathway

The PI3K-AKT signaling pathway is crucial for stem cell survival, proliferation, and apoptosis [102]. Notably, key proteins within this pathway are closely regulated by m6A modification [103]. For instance, *METTL3*-mediated m6A modification directly modulates the PI3K-AKT pathway. Knockdown of *METTL3* inhibits the phosphorylation of *AKT* (also known as RAC-alpha serine/threonine protein kinase), thereby suppressing *PI3K*/*AKT* activation and subsequently inhibiting glycolysis in stem cells [104]. Furthermore, reduced expression of *METTL3* and *EPPK1* (epiplakin 1) [105] can upregulate *PI3K* (phosphatidylinositol 3-kinase) expression, collectively disrupting stem cell homeostasis [106].

**Figure 5:**
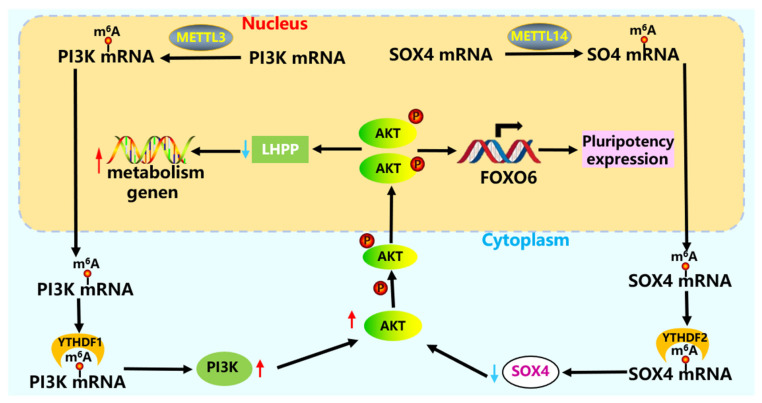
m^6^A modification participates in the regulation of the PI3K-*AKT* pathway in stem cells. *METTL3* and *YTHDF1/2* regulate the expression of P13K and SOX4 proteins in the PI3K-*AKT* pathway, thereby activating the upregulation of *AKT* expression [104,108]. After *AKT* is phosphorylated, it enters the nucleus, promoting the expression of *FOXO6* and metabolism-related genes, enhancing stem cell pluripotency, and stabilizing energy metabolism levels [112].

Overexpression of *METTL14* increases the m^6^A enrichment of *PTEN* (phosphatase and tensin homolog), promoting *PTEN* expression. The increased expression of PTEN significantly inhibits the activation of the PI3K/*AKT* pathway [107] (Figure 5). *METTL14* affects the expression of transcription factor *SOX4* (sex-determining region Y-box transcription factor 4), and the degradation of *SOX4* mRNA depends on *YTHDF2.* Low levels of SOX4 increase the expression levels of *PI3K* and *AKT* proteins, activate glycolysis in stem cells, and have a certain inhibitory effect on the malignant process of CRC [108]. Lowering the expression of *YTHDF1* significantly reduces key proteins in the PI3K/*AKT* signaling pathway, such as *PI3K*, *AKT*, and *mTOR* (mammalian target of rapamycin). YTHDF1 mainly regulates the translation of *AKT2* and *AKT3* rather than transcription [109,110]. Low levels of *YTHDF2* significantly increase the expression of *LHPP* (phospholysine phosphohistidine inorganic pyrophosphate phosphatase) and growth inhibitor *NKX3-1* (NK3 homeobox 1), inhibit *AKT* phosphorylation, and significantly suppress the proliferation and migration of PCa cells. At the same time, reduced *AKT* phosphorylation levels affect the expression of glycolytic genes and the maintenance of stem cell pluripotency [111]. *FTO*, as a downstream target of *PI3K*-*AKT*, activates *PI3K-AKT* through a transcription factor *FOXO6* (forkhead box protein O 6), affecting the energy metabolism and pluripotency of stem cells [112].

### 6.3. m^6^A Modification and JAK-STAT Signaling Pathway

**Figure 6:**
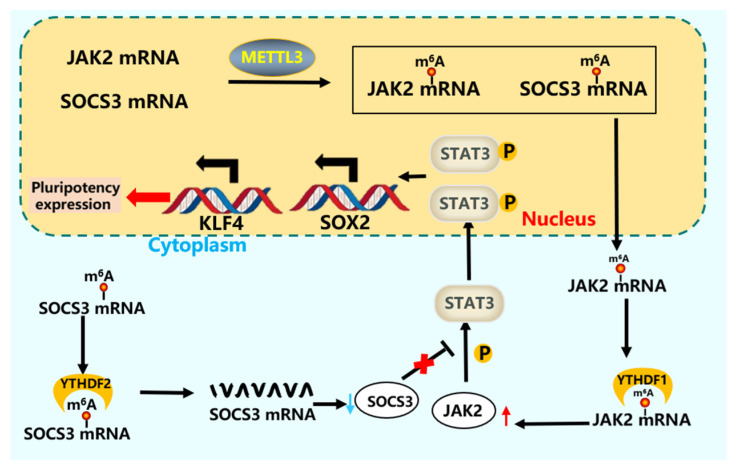
m^6^A modification regulates the *JAK-STAT* pathway in stem cells [116]. *YTHDF1* and *YTHDF2* recognize the modification of *JAK2* and *SOCS3* by *METTL3*, promote *JAK2* mRNA expression and *SOCS2* mRNA degradation, enhance *STAT3* protein expression, phosphorylate *STAT3* protein translocation into the nucleus, increase *SOX2* and *KLF4* expression, and enhance stem cell pluripotency [16].

The JAK-STAT pathway regulates the proliferation and apoptosis of stem cells [113], and the expression of key proteins in the pathway is regulated by m^6^A modification at the transcriptional level [94]. *METTL3* directly modifies the *JAK1* (just another kinase) mRNA, while *METTL3* and *YTHDF2* maintain the stability of *JAK1* mRNA [114]. After m^6^A modification, the JAK-STAT signal directly participates in the regulation of iPSCs [16]. *METTL3* maintains the pluripotency of iPS cells by mediating the expression of *JAK2*-*STAT3*. *KLF4* is a direct downstream target of *JAK*-*STAT3*, which is activated by phosphorylated *STAT3* (signal transducer and activator of transcription 3) and indirectly activates *SOX2*. Overexpression of *METTL3* directly enhances the phosphorylation level of *STAT3* and increases the expression of *SOX2* and *KLF4* [16,115]. *SOCS3* (suppressor of cytokine signaling 3) is a key negative regulator of the *JAK2*-*STAT3* pathway. *METTL3* modifies *JAK2* and *SOCS3*, and *JA*K2 and *SOCS3* are targets of *YTHDF1* and *YTHDF2*, respectively. Decreasing the m^6^A modification of *JAK2* and *SOCS3* inhibits *YTHDF1*-mediated *JAK2* translation, blocks *YTHDF2*-dependent *SOCS3* mRNA decay, and impairs the pluripotency of iPSCs [16] (Figure 6).

### 6.4. m^6^A Modification and Hippo Signaling Pathway

The Hippo pathway directly participates in the complex biological processes of stem cells and plays an important role in their proliferation, self-renewal, and differentiation [117]. m^6^A modification regulates the Hippo signaling pathway through key regulatory factors such as *LATS1*, *YAP*, and *TAZ* [94]. m^6^A modification directly affects the translation of *YAP1* mRNA, regulating the proliferation and differentiation of stem cells [118].

**Figure 7:**
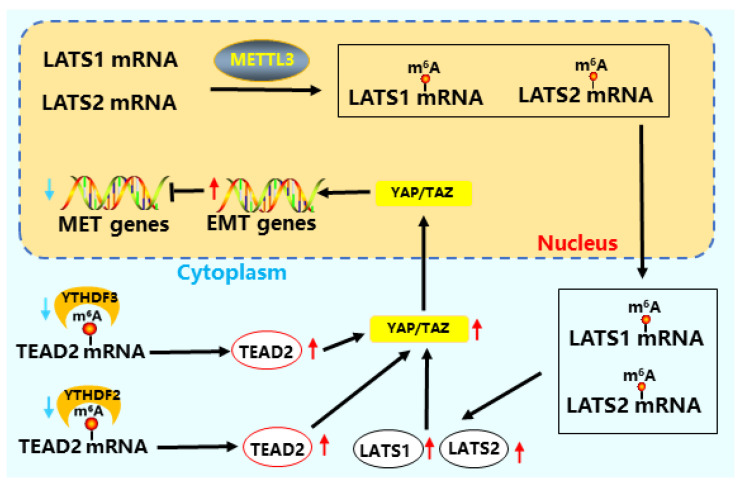
m^6^A modification regulates the Hippo pathway in stem cells. *METTL3* promotes *LATS1/2* expression by modifying *LATS1/2* mRNA [119,120], leading to an increase in *YAP/TAZ* expression. At the same time, reducing the expression of *YTHDF2/3* and increasing the expression of *TEAD2* also leads to an increase in *YAP/TAZ*, which enhances the expression of EMT-related genes and inhibits the expression of MET-related genes [61,126].

The increase in *METTL3* expression directly activates the Hippo pathway and regulates *YAP* nuclear translocation, affecting the proliferation and differentiation of CRC cells through lncRNA [119,120]. *LATS1* directly regulates the phosphorylation level of *YAP/TAZ*, increases METTL3 or decreases *YTHDF2* expression, and activates *YAP/TAZ* in the Hippo signaling pathway [121] (Figure 7). The combination of *YTHDF2* and *YAP* mRNA reduces the stability of mRNA, while *YTHDF2* recruits *AGO2* (argonaute RISC catalytic component 2) to promote the degradation of YAP mRNA, affecting the production of related tumor factors and inhibiting the occurrence of tumors in breast cancer [122,123]. *YTHDF2/3* regulates somatic reprogramming by regulating the relationship between the Hippo pathway and EMT [61]. *YTHDF2/3* recruits the disenergizing enzyme complex CCR4-NOT and PAN2/PAN3 to degrade TEAD2 mRNA, reduce *TEAD2* expression, promote *YAP/TAZ* nuclear translocation and MET [124,125,126]. The increased expression of *YTHDF2/3* promotes *YAP* nuclear translocation, promotes the expression of pluripotent factors, and accelerates somatic reprogramming [127].

In summary, key components of signaling pathways such as Wnt, PI3K-AKT, *JAK-STAT*, and Hippo, after being modified with m^6^A, play a series of regulatory roles in stem cells. These signaling pathways are directly involved in the regulation of somatic reprogramming (Table 1).

### 6.5. m^6^A Modification: A Core Integrator of Stem Cell Signaling Network Crosstalk

The Wnt, PI3K-AKT, JAK-STAT, and Hippo pathways do not operate independently but form a dense regulatory network that collectively determines stem cell fate. m^6^A modification may play a central role in integrating signals and amplifying effects by simultaneously targeting key nodes across multiple pathways. Wnt-PI3K-AKT Synergy: These two pathways engage in well-known crosstalk. On the one hand, the PI3K-AKT pathway stabilizes *β-catenin* by phosphorylating and inhibiting *GSK-3β*, thereby positively reinforcing Wnt signaling. This review highlights that m^6^A degradation of *GSK-3β* mRNA via *YTHDF2* [101] and downregulation of *APC* expression through the *METTL3-YTHDF* axis [84] both lead to *β-catenin* accumulation. This suggests m^6^A may simultaneously weaken two major negative regulatory mechanisms of *β-catenin* (*APC* and *GSK-3β*), synergistically amplifying Wnt pathway output. Transcriptional Cooperation Between Hippo and Wnt: Upon nuclear translocation, Hippo downstream effectors *YAP/TAZ* not only bind to *TEAD* family transcription factors but also interact with Wnt pathway effectors TCF/LEF, jointly regulating an overlapping set of target genes (e.g., *CYR61* and *CTGF*). This review indicates that m^6^A influences *YAP* activity by degrading *Tead2* mRNA via *YTHDF2/3* [61,126]. This suggests m^6^A may indirectly regulate the cooperative efficiency between YAP/TAZ and Wnt pathway effectors by modulating the Hippo pathway, thereby broadly impacting gene programs controlling proliferation and pluripotency.

These interactions demonstrate that m^6^A’s regulation of individual signaling molecules (e.g., *GSK-3β*, *APC*, and *Tead2*) amplifies and integrates effects through networked crosstalk, ultimately coordinating global regulation of stem cell self-renewal, differentiation, or reprogramming. Future studies should increasingly employ systems biology approaches to map the coupling between the m^6^A modification landscape and the dynamics of stem cell signaling networks.

Established Node-Specific Regulation: There is strong evidence that m^6^A can modulate individual key nodal proteins across major pathways (e.g., *APC* in Wnt and *PTEN* in PI3K-AKT) [84,107]. This establishes m^6^A as a bona fide post-transcriptional regulator of signaling cascades.

The “Integration” Caveat: Most examples show m^6^A acting on parallel, independent nodes. True network integration would require evidence that a single m^6^A event simultaneously coordinates the activity of two or more pathways in a coordinated manner (e.g., via a shared regulator like *GSK-3β*). Such higher-order coordination is suggested but not definitively proven in stem cell fate decisions.

Critical Disconnect between Models: A profound gap exists between the detailed oncogenic signaling roles of m^6^A in cancer stem cells (CSCs) and the understanding of its function in physiological stem cell signaling during development or tissue repair. It is unclear whether the aggressive, pro-growth wiring seen in CSCs reflects a hijacking or an amplification of normal stem cell mechanisms. Bridging this gap is essential for therapeutic safety.

## 7. m^6^A Modification and Somatic Reprogramming

Somatic reprogramming is an integrative paradigm. The complete conversion of a somatic cell to a pluripotent state requires the synchronized rewiring of all regulatory layers discussed thus far. This section uses reprogramming as a model process to illustrate the stage-specific, coordinating function of m^6^A. We will delineate how, in a temporally ordered manner, m^6^A (1) initiates dedifferentiation and metabolic reprogramming (leveraging mechanisms from Section 3 and Section 5); (2) regulates the EMT/MET balance and consolidates the pluripotency network (integrating controls from Section 3, Section 4 and Section 6); and (3) finalizes mitochondrial and epigenetic remodeling. This analysis showcases m^6^A not as a collection of parts, but as a unified system driving complex state transitions.

Somatic reprogramming refers to the reprogramming of terminally differentiated cells into iPSCs [137]. This process is typically initiated by the co-expression of key transcription factors, notably *OCT4*, *SOX2*, *KLF4*, and *c-MYC* [138]. Successful reprogramming entails a coordinated cascade of events, including the silencing of somatic genes, a metabolic switch from oxidative phosphorylation to glycolysis (OGS), a mesenchymal-to-epithelial transition (MET), and the activation of the core pluripotency network [139]. Given the profound epigenetic remodeling required, the regulation of epigenetic modifications, such as m^6^A, is a critical determinant of reprogramming efficiency [140]. m^6^A modification, as a key layer of epigenetic transcriptional regulation, plays a core, temporally and stage-specific role at different phases of this process. By precisely regulating transcription factors, cellular state transitions, and energy metabolism, it synergistically drives cells toward reprogramming into a pluripotent state. Following initiation in the early stage, cells must first disrupt existing somatic programs and activate metabolic shifts supporting pluripotency acquisition. The core task of the intermediate stage involves activating the central pluripotency transcription factor network and completing metabolic reprogramming (MET) to establish stable epithelial-like iPSC characteristics. In the late stage of reprogramming, cells must consolidate the pluripotent epigenetic state and complete mitochondrial function remodeling to achieve full establishment and maintenance of pluripotency.

### 7.1. Early Phase: Initiation, Dedifferentiation, and Metabolic Reprogramming

The m^6^A machinery precisely orchestrates the expression of core pluripotency factors to facilitate reprogramming. Writers such as *METTL3* and *METTL14* create a permissive epigenetic landscape: *METTL3* broadly promotes miRNA expression [141] and, via the mediator *ZFP217*, upregulates *OCT4*, *SOX2*, and *NANOG* [19]; meanwhile, *METTL14* overexpression induces a senescence-associated secretory phenotype (SASP), whose component *IL-6* enhances iPSC induction efficiency [142]. Subsequently, readers *YTHDF2* and *YTHDF3* execute the clearance of somatic program mRNAs (e.g., *Tead2* and *Tgfb1*) by recruiting the CCR4-NOT and PAN2-PAN3 deadenylase complexes, respectively, thereby removing barriers to reprogramming [61,124,125]. Collectively, through these writer- and reader-mediated actions, m^6^A modification exerts central control over the pluripotency transcription factor network, including *OCT4*, *SOX2*, *Nanog*, and *KLF4* (Table 2).

### 7.2. The Role of m^6^A Modification in EMT

The dynamic balance between epithelial–mesenchymal transition (EMT) and its reverse process, mesenchymal–epithelial transition (MET), is crucial for somatic reprogramming [145,146] (Figure 4). Early reprogramming involves a transient EMT phase, which activates epigenetic modifiers like *Bmi1* and *Ezh2* [147]. Subsequently, the establishment of MET is essential; it is characterized by the downregulation of *TGF-β*, *Snail*, and *Zeb* family members [145].

**Figure 8:**
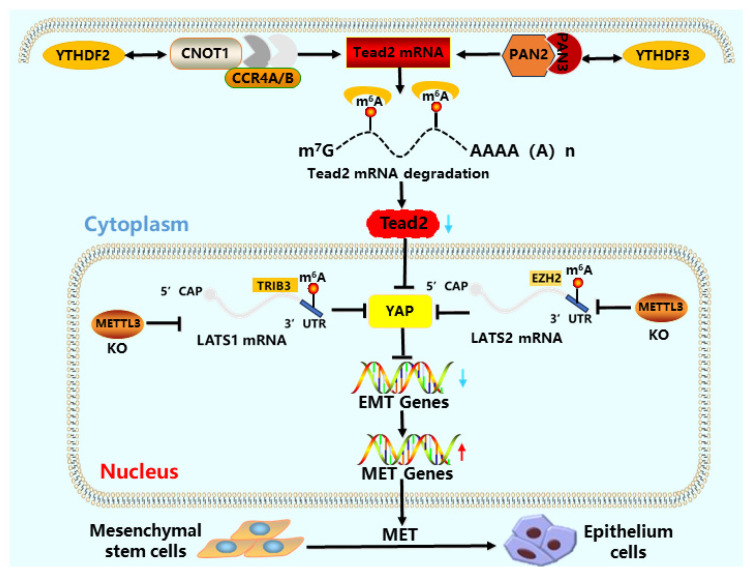
Somatic reprogramming requires a transition from a mesenchymal to an epithelial state (MET). This figure shows how m^6^A regulates this balance at different stages. (Pathway A, via the Hippo pathway): During the mid-late stages of reprogramming, m^6^A readers *YTHDF2/3* promote MET and facilitate reprogramming by degrading *Tead2* mRNA, which inhibits the activity of the Hippo effector YAP and consequently downregulates EMT genes [61,124,125]; (Pathway B, via the methyltransferase): Loss of *METTL3* inhibits *LATS1/2* expression, also leading to decreased *YAP* activity and promotion of MET [119,120]. These two seemingly independent regulations may function at different time points or in different cell subpopulations, working synergistically to ensure the timely occurrence of MET.

The m^6^A modification system acts as a key regulator of this EMT-MET equilibrium. It directly targets core EMT regulators: for instance, *METTL3* and *YTHDF1* control the translation of *Snail* mRNA, thereby influencing EMT/MET dynamics [148]. Furthermore, the m^6^A reader proteins *YTHDF2* and *YTHDF3* promote the MET phase and enhance reprogramming efficiency by degrading *Tead2* mRNA. This reduction in TEAD2 levels prevents nuclear accumulation of *YAP/TAZ*, a key event that otherwise hinders MET [61,126] (Figure 8). Thus, through coordinated actions on multiple nodes, m^6^A finely tunes the EMT-MET switch to facilitate reprogramming.

### 7.3. m^6^A Modification and Energy Metabolism in Somatic Reprogramming

m^6^A modification plays a pivotal role in reprogramming by orchestrating the essential metabolic switch and modulating mitochondrial function [18,48,77]. A core event is the oxidative phosphorylation to glycolysis (OGS) transition. This process is initiated by *ERRα/γ*-activated OXPHOS and subsequently driven by a shift towards glycolysis [149,150,151]. In addition, the expression of *ERRα* is regulated by *HIF-1α* (hypoxia inducible factor-1α) [152]. *HIF-1α* directly modifies glycolytic kinases such as *PDK1*, *PKM2*, and *HK2* to promote OGS and improve the efficiency of somatic reprogramming [153,154]. *HIF-1α* is regulated by *YTHDF1*, which works together with *ATG2A* (autophagy-related 2A) and *ATG14* (autophagy-related 14) to regulate *HIF-1α* [155]. Activated glycolysis enhances cellular acetyl-CoA and lactate levels, enhances H3K27ac (acetyl histone H3 Lys27) and lactylation of H3K18la (histone 3 on lysine residue 18) at pluripotent gene loci, and promotes somatic reprogramming [91].

**Figure 9:**
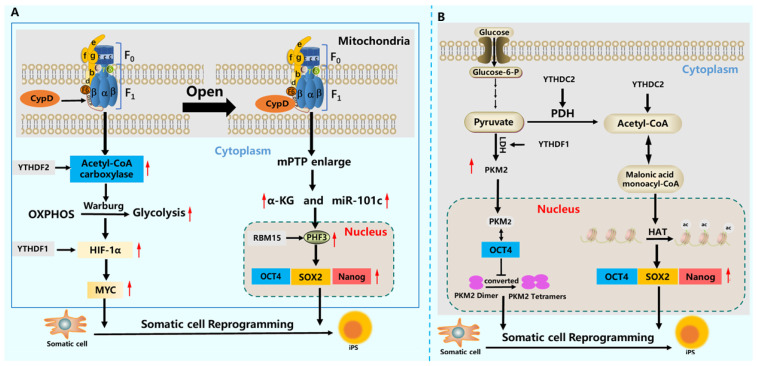
The role of m^6^A modification in mitochondrial dynamics and glycolysis in somatic reprogramming. (**A**) m^6^A modification and mitochondrial dynamics. m^6^A modification is involved in the control of mitochondrial ATP synthase, which leads to mitochondrial permeability opening [161,162]. The expression of α-KG and *miR-101c* increases, activating the expression of *PHF8* and promoting the expression of multifunctional factors such as *OCT4*, *SOX2*, and *Nanog* [157]; *YTHDF2* promotes the upregulation of acetyl-CoA carboxylase expression, enhances Warburg, increases *HIF-1α* and *MYC* content, and promotes reprogramming efficiency [83]. (**B**) m^6^A modification and glycolysis. m^6^A modification controls the occurrence of glycolysis and the expression of acetyl-CoA [91]. Elevated pyruvate promotes an increase in *PKM2* expression, and *PKM2* and *OCT4* work together to improve reprogramming efficiency [153]; *YTHDC2* increases acetyl-CoA levels, promotes the functional conversion of histone acetyltransferase, and increases the expression of multifunctional factors such as *OCT4*, *SOX2*, and *Nanog*, affecting reprogramming efficiency [160].

Mitochondrial physiology, encompassing dynamics and the permeability transition pore (mPTP), is critically regulated by m^6^A and directly influences somatic reprogramming [156,157,158,159] (Figure 9A,B). m^6^A supports the metabolic reprogramming of cells by enhancing mitochondrial oxidative capacity. It promotes acetyl-CoA production, a key substrate for histone acetylation: *YTHDC2* stabilizes *PDHA1* mRNA to boost nuclear acetyl-CoA levels [79,160]. This aligns with the role of ATP synthase in coupling metabolic shifts to reprogramming [161], while *METTL3* can regulate mitochondrial ATP synthase activity [162]. In parallel, m^6^A exerts precise control over the mPTP, a pivotal gateway in mitochondrial signaling. The opening of the mPTP elevates mitochondrial reactive oxygen species (mtROS), which in turn activates (via *miR-101c*) the histone demethylase *PHF8*. *PHF8* erases repressive histone marks (H3K9me2/H3K27me3), thereby facilitating epigenetic reprogramming [76,157] (Figure 9B). *METTL3* contributes to this regulatory layer by controlling the expression of the mPTP-associated transcription factor *NRF1* [158]. Therefore, through coordinated regulation of mitochondrial metabolism and permeability-dependent signaling, m^6^A modification plays an essential role in establishing the mitochondrial conditions necessary for somatic reprogramming.

Well-Defined Stage-Specific Functions: The field has reached a consensus on the temporal sequence of m^6^A actions: early somatic gene silencing (via *YTHDF2/3*), mid-phase MET promotion, and late-stage metabolic/epigenetic consolidation [61,124,125]. This provides a refined view beyond simple “pro-” or “anti-” reprogramming effects.

The In Vitro Bottleneck: This exquisite mechanistic understanding is almost entirely confined to fibroblast reprogramming in culture. A major, unresolved question is whether this same playbook operates during in vivo reprogramming (e.g., in injury models) or in reprogramming clinically relevant human somatic cells (e.g., blood or hepatocytes), which may have distinct m^6^A landscapes and dependencies.

Therapeutic Translation Challenge: The biggest controversy lies in the application. While modulating m^6^A can enhance iPSC generation in vitro, the potential risks of altering such a fundamental regulatory layer in vivo (e.g., for in situ regeneration) are immense and unknown. The field must confront the trade-off between efficiency and safety, moving from proof-of-concept to clinically viable strategies.

## 8. Current Challenges and Future Perspectives

Based on the integrated perspective of this review, we arrive at a core conclusion: the strong context dependence of m^6^A modification not only reflects its complexity but also constitutes its functional essence as a precise regulator of stem cell fate. The varying, sometimes opposing, outcomes described for factors like *METTL3* or *YTHDF2* across different stem cell systems (Section 3, Section 6 and Section 7) are logical consequences of their roles within specific molecular contexts. This framework forces a shift from asking “What does protein X do?” to “What does protein X do, in which cell state, and in response to what signals?”

The methyltransferase *METTL3* illustrates this principle. In mouse embryonic stem cells (mESCs) and induced pluripotent stem cells (iPSCs), *METTL3* primarily supports pluripotency by stabilizing transcripts of core factors such as *Nanog* and facilitating the silencing of somatic genes during reprogramming [16,53]. In stark contrast, within cancer stem cells (CSCs) of various malignancies—including leukemia, gastric, and pancreatic cancer—*METTL3* often functions as an oncogene. It promotes unlimited proliferation, metabolic reprogramming (e.g., enhanced glycolysis), and drug resistance by activating pro-survival pathways like Wnt/*β-catenin* and PI3K/*AKT* [84,104]. This functional duality likely originates from cell-type-specific RNA targeting and interactions with distinct upstream signals and epigenetic landscapes. A similar context-dependent pattern is observed for reader proteins. *YTHDF2* can promote induced pluripotency by degrading differentiation-related mRNAs (e.g., *Tead2*) [61], yet it acts as a tumor suppressor in prostate cancer by destabilizing oncogenic transcripts [111]. Likewise, the demethylases *ALKBH5* and *FTO* fine-tune pluripotency in ESCs but are frequently upregulated in glioblastoma or leukemia stem cells to drive self-renewal, block differentiation, and aid immune evasion [24,68]. This profound heterogeneity underscores a critical caveat: broad statements such as “*METTL3* promotes stemness” or “*YTHDF2* inhibits stemness” are oversimplified and potentially misleading. Consequently, therapeutic strategies targeting the m^6^A pathway must be designed to selectively disrupt pathological modifications in diseased cells while preserving the physiological homeostasis of normal stem cells.

The functional output of m^6^A is shaped by the intrinsic nature of the stem cell. Embryonic stem cells (ESCs) employ m^6^A as a molecular “rheostat” to enable state transitions, such as priming for differentiation by destabilizing naïve pluripotency transcripts [49]. Adult somatic stem cells, however, often utilize m^6^A to maintain homeostasis within their niche, precisely regulating fate decisions in response to local cues [74]. Cancer stem cells (CSCs) represent a pathological adaptation, hijacking the m^6^A machinery to enforce a plastic, pro-survival state that fuels tumorigenesis, therapy resistance, and metastasis [18]. Recognizing this spectrum of physiological versus pathological roles is fundamental for developing precise interventions.

Significant debate persists regarding the functions of cytoplasmic m^6^A readers, particularly the YTHDF family (*YTHDF1/2/3*). Early models assigned specialized roles—translation promotion (*YTHDF1*), decay mediation (YT*HDF2*), and cooperative function (*YTHDF3*) [33,125]. Recent evidence, however, suggests considerable redundancy. During somatic reprogramming, for instance, the combined knockdown of *YTHDF2* and *YTHDF3*, but not single knockdowns, is necessary to block the degradation of specific mRNAs like *Tead2* [61,124]. Conversely, other contexts reveal clear specialization; in porcine iPSCs, *YTHDF1* specifically regulates JAK2 translation, while *YTHDF2* uniquely controls *SOCS3* mRNA decay [16]. Resolving this redundancy-specificity paradox will require integrated approaches, including simultaneous multi-gene knockout, spatial proteomics, and single-cell analyses, to define condition-specific reader complexes and their precise targets.

A major translational challenge lies in bridging the gap between robust in vitro mechanistic findings and their in vivo validation. Most current knowledge derives from cultured stem cells or cancer lines, which, while indispensable for mechanistic dissection, cannot fully capture the complexity of native niches, systemic metabolism, and immune interactions. For example, while the role of *YTHDF2/3* in reprogramming is well-established in vitro [61], their functions in adult stem cell homeostasis in vivo remain less clear. Similarly, although *METTL3* inhibitors show promise in suppressing CSC growth in culture and xenograft models [22], their impact on normal tissue regeneration and long-term safety is largely unknown. Future research must prioritize physiologically relevant models—such as genetically engineered animals and patient-derived organoids—to validate mechanisms and rigorously assess the potential on-target toxicities of perturbing this fundamental RNA regulatory pathway.

## 9. Conclusions

Stem cells possess the capacity for self-renewal and multipotent differentiation, with their functional changes regulated by multiple factors, including transcription factors, histone modifications, and energy metabolism.

As the most abundant RNA modification in eukaryotic cells, m^6^A plays a huge role in almost every aspect of biological regulation by regulating RNA modification in different ways. At the same time, we are gradually mastering the functions of m^6^A modification and the key regulatory factors of methylation and demethylation. The dynamic regulation of m^6^A modification is a new post-transcriptional regulation mechanism, which is closely related to the regulation of stem cell pluripotency maintenance, histone modification, energy metabolism, and reprogramming. Energy metabolism directly affects the pluripotency, proliferation, and differentiation of stem cells. Histone modification regulates the pluripotency and differentiation of stem cells by regulating the opening and closing of chromatin. Pluripotency maintenance, histone modification, and energy metabolism are all involved in regulating somatic reprogramming. m^6^A modification is involved in the regulation of signaling pathways such as Wnt, PI3K-AKT, JAK-STAT, and Hippo, which directly regulate stem cell function and somatic reprogramming. The evidence synthesized through this integrative framework reveals that m^6^A regulation is fundamentally context-dependent and multi-layered. The apparent contradictions, such as *METTL3* promoting pluripotency in ESCs but oncogenesis in CSCs, are resolved when viewed through the lens of distinct cellular “contexts”—different target RNA repertoires, signaling environments, and epigenetic landscapes. This underscores that m^6^A machinery components are not simply “on/off” switches for stemness but versatile tools whose functional output is defined by the system in which they operate. Future research must therefore prioritize defining these contexts through integrated omics approaches and sophisticated in vivo models. By doing so, we can move from a phenomenological catalog of m^6^A functions towards a predictive understanding of its role in fate decisions, unlocking its precise therapeutic potential in regeneration and disease. These studies reveal a fascinating but unexplored regulation of the interaction between gene expression and protein translation and the need for coordination with other regulatory networks. To move forward, the field must rigorously address the translational gap between in vitro mechanisms and in vivo physiology, leveraging advanced models to unlock the true therapeutic potential of targeting m^6^A in regenerative medicine and oncology.

Although the m^6^A-targeted modification of stem cells has shown broad prospects in the medical field, there are still challenges in related research. These related studies are still in their infancy, and many potential mechanisms have not yet been discovered. More in vivo studies and clinical trials are urgently needed to confirm the potential clinical significance of m^6^A modification in stem cell therapy, which will significantly improve the effectiveness of stem cell therapy in the treatment of human diseases.

In different types of stem cells, m^6^A modification affects the biological behavior of stem cells by altering the stability of key mRNA involved in stem cell function regulation and by activating or inhibiting related signaling pathways. Therefore, further exploration of the regulatory mechanisms of m^6^A modification in stem cell function and somatic reprogramming will provide great prospects for research in regenerative medicine, disease treatment, and drug screening.

## Figures and Tables

**Table 1:** Regulatory effects of different signaling pathways on somatic reprogramming.

Signal Pathway	Mechanisms for Regulating Reprogramming	References
Wnt	After activation, *β-catenin* binds to the effector factor TCF to maintain the expression of pluripotent genes, such as *SOX2*. The efficiency of reprogramming is regulated by the binding of *β-catenin* and repressor protein TCF7L1.	[128,129]
PI3K-AKT	The pluripotent factor *SOX2* is a direct target of phosphorylated *AKT*, and phosphorylated *AKT* directly regulates the expression of *SOX2*, regulating the induction efficiency of iPS cells. Activated *AKT* can replace bFGF and improve reprogramming efficiency.	[130,131]
JAK-STAT	Activated *STAT3* interacts with *Nanog* or KLF4, respectively, to improve reprogramming efficiency. In the later stage of reprogramming, *STAT3* activates the endogenous *OCT4* gene, improving reprogramming efficiency.	[132,133]
Hippo	*LATS2* inhibits reprogramming by antagonizing TAZ factors. Reducing the expression of *LATS2* promotes the nuclear translocation of *YAP*. After translocation, *YAP* regulates reprogramming by interacting with *OCT4* and *SOX2* and combines with *TEAD* alone to regulate reprogramming.	[134,135,136]

**Table 2:** Regulation of m^6^A modification on reprogramming transcription factors and pluripotent factors.

m^6^A Modifier	Transcription Factors	Molecular Mechanism	Reference
*METTL3*	*Nanog*	*METTL3-METTL14*-WTAP complex binds with *SMAD2/3* to regulate Nanog levels and cellular pluripotency	[54]
	*SOX2*	*METTL3* deficiency interferes with the expression of *JAK2* and *SOSC3*, inactivates the *JAK2* pathway, blocks *SOX2* transcription, and inhibits piPSC differentiation	[16]
	*KLF4*	*METTL3* deficiency interferes with *JA*K2 and *SOSC3* expression, blocks *KLF4* transcription, and inhibits piPSC differentiation	[16]
*METTL14*	*OCT4*	Overexpression of *METTL14* increases the expression of *OCT4* and improves reprogramming efficiency	[125]
*ALKBH5*	*Nanog*	In the late stage of reprogramming, overexpression of *ALKBH5* improves reprogramming efficiency by stabilizing the transcript of *Nanog*	[143]
*YTHDF1*	*OCT4*	RNA/DNA binding protein binds to *OCT4* and *YTHDF1* promoters to regulate hiPSC differentiation	[144]

## Data Availability

No new data were created or analyzed in this study.
